# Comparison of genotype clustering tools with rare variants

**DOI:** 10.1186/1471-2105-15-52

**Published:** 2014-02-21

**Authors:** Louis-Philippe Lemieux Perreault, Marc-André Legault, Amina Barhdadi, Sylvie Provost, Valérie Normand, Jean-Claude Tardif, Marie-Pierre Dubé

**Affiliations:** 1 Beaulieu-Saucier Université de Montréal Pharmacogenomics Center, Montreal Heart Institute Research Center, 5000 Bélanger Street, Montréal, Canada; 2 Université de Montréal, Faculty of Medicine, 2900 chemin de la tour, Montréal, Canada

## Abstract

**Background:**

Along with the improvement of high throughput sequencing technologies, the genetics community is showing marked interest for the *rare variants/common diseases* hypothesis. While sequencing can still be prohibitive for large studies, commercially available genotyping arrays targeting rare variants prove to be a reasonable alternative. A technical challenge of array based methods is the task of deriving genotype classes (homozygous or heterozygous) by clustering intensity data points. The performance of clustering tools for common polymorphisms is well established, while their performance when conducted with a large proportion of rare variants (where data points are sparse for genotypes containing the rare allele) is less known. We have compared the performance of four clustering tools (*GenCall*, *GenoSNP*, *optiCall* and *zCall*) for the genotyping of over 10,000 samples using the Illumina’s HumanExome BeadChip, which includes 247,870 variants, 90% of which have a minor allele frequency below 5% in a population of European ancestry. Different reference parameters for *GenCall* and different initial parameters for *GenoSNP* were tested. Genotyping accuracy was assessed using data from the *1000 Genomes Project* as a gold standard, and agreement between tools was measured.

**Results:**

Concordance of *GenoSNP*’s calls with the gold standard was below expectations and was increased by changing the tool’s initial parameters. While the four tools provided concordance with the gold standard above 99% for common alleles, some of them performed poorly for rare alleles. The reproducibility of genotype calls for each tool was assessed using experimental duplicates which provided concordance rates above 99%. The inter-tool agreement of genotype calls was high for approximately 95% of variants. Most tools yielded similar error rates (approximately 0.02), except for *zCall* which performed better with a 0.00164 mean error rate.

**Conclusions:**

The *GenoSNP* clustering tool could not be run straight “out of the box” with the HumanExome BeadChip, as modification of hard coded parameters was necessary to achieve optimal performance. Overall, *GenCall* marginally outperformed the other tools for the HumanExome BeadChip. The use of experimental replicates provided a valuable quality control tool for genotyping projects with rare variants.

## Background

More than 13,500 genome-wide association studies (GWAS) of complex human diseases have been performed since 2008 [1]. The majority of GWAS were conducted using common single nucleotide polymorphism (SNP) arrays targeting markers that were identified from the international HapMap project [2-4]. These studies are based on the assumption that common traits are driven by common low-penetrance polymorphisms with a frequency of more than one or five percent in the population [5]. A vast proportion of the heritability of complex traits remains unexplained [6]. However, advances in genomic technologies now allow for the search of rare variants of modest to intermediate penetrance [7].

SNP arrays offer the possibility of rapid genotyping of thousands of samples with highly reliable results at low cost. Several commercial arrays now include a large fraction of rare single nucleotide variants (SNV) discovered by high-throughput sequencing technologies. The latter, while still expensive compared to SNP arrays, allows for the discovery of all variants, rare and common, located in the genome of sequenced individuals. The Illumina HumanExome BeadChip provides a compromise between genotyping SNP arrays and next generation sequencing by enabling the genotyping of rare SNVs in thousands of samples at relatively low cost. The HumanExome BeadChip is enriched for rare and low frequency coding variations previously identified from the sequenced exomes of approximately 12,000 individuals of diverse populations for variations seen in more than two individuals and in more than two sequencing projects [8]. Compared to other genotyping platforms targeting millions of markers, the proportion of rare variants (minor allele frequency <5%) included in the HumanExome BeadChip is considerably larger.

A recent review of clustering tools for widely used Illumina BeadChip arrays was presented by Ritchie *et al.*[9]. They reported that some tools performed marginally better than others for common and rare variants (lowest frequency around 0.05). The authors noted that methods borrowing information from other SNPs (*e.g.within-sample* information) to genotype rare variants could theoretically outperform reference-based methods, as these would suffer from the limited information available in the training sets for the homozygous and heterozygous clusters of rare alleles. Such *within-sample* methods are implemented in *GenoSNP*[10]. Some tools, such as *M3*[11] and *optiCall*[12], use a mixture of *between-* and *within-sample* approaches. Other tools, such as *GenCall* (available in the *GenomeStudio* software) [13], rely on a reference cluster file to cluster marker genotypes one at a time. The *zCall* tool exclusively genotypes markers that have been previously “missed” by a another tool, and was also recently described [14].

For this project, the performance of *GenCall*, *GenoSNP*, *optiCall* and *zCall* for clustering markers from the HumanExome BeadChip have been analysed and compared. With the growing interest of the community for studies with rare variants [5,6,15,16], this “head to head” comparison will provide guidance for study design, tool selection and results interpretation.

## Methods

### Clustering tools

Four clustering tools were compared: *GenCall*, part of the *GenomeStudio* software version V2011.1 [13], *GenoSNP* version 1.3 [10], *optiCall* version 0.3.3 [12] and *zCall* version 3.2 [14]. All four tools differ with respect to their genotype calling method.

#### GenCall (GenomeStudio)

*GenCall* is Illumina’s proprietary tool and is available through the *GenomeStudio* software. For a complete description of this tool, refer to Ritchie *et al.*[9]. In brief, this tool uses the normalized microarray intensities for both alleles (noted *X* and *Y*) to compute the associated polar coordinates (*r* and *θ*) for each marker/sample pair. Next, using a *between-sample* model, meaning that it calls one marker by looking at the population of samples, it assigns genotypes by determining the nearest cluster using a reference containing the expected position of each genotype cluster for every marker as determined from the HapMap data. If required, the user may modify the position of each of the expected clusters to be more representative of the data at hand. By pre-assigning the expected position of each cluster, this method can readily provide a genotyping assignation of rare variants for studies having only a small number of samples. However, due to experimental variabilities and genomic variations in different populations, the position of the observed cluster’s centroid might shift when compared with the expected one. A considerable amount of manual cluster adjustments might be needed to achieve good genotype calls.

For this project, a custom cluster file was created by modifying the expected position of the genotype cluster’s centroid for a subset of markers by using all samples from the dataset. The markers selected for manual inspection were: (1) markers with a high heterozygous frequency, (2) markers with a low mean intensity, (3) markers with a low call frequency, (4) markers with a low minor allele frequency with no heterozygous calls, (5) markers showing an excess of heterozygous calls, (6) markers with low AA T means or low BB T means^a^, (7) mitochondrial markers, (8) markers on sex chromosomes, (9) markers that fail reproducibility tests, (10) markers with a small cluster separation or (11) markers with low quality score. To compare the efficiency of this modified cluster file, the results from *GenCall* with the original cluster file were also included in the analysis.

#### GenoSNP

*GenoSNP* uses a *within-sample* model, meaning that it assigns genotypes to all markers of a single sample at once [10]. It uses raw *X* and *Y* allele intensities extracted using the *GenomeStudio* software and calls genotypes by fitting a four-components mixture of Student’s *t*-distributions on different subsets of markers (separated by Bead Pools) by the mean of a Variational Bayes Expectation Maximization algorithm (VB-EM). The use of this method improves robustness by allowing uncertainty in the statistical model, in contrast to standard expectation maximisation methods. *GenoSNP* computes the posterior probability of the marker genotype calls. As this tool calls one sample at a time, it offers the flexibility of providing final sample genotyping results before the whole study dataset is ready to be processed. It can also be parallelized by running the tool on multiple samples at a time, and it does not require a reference panel. It is generally expected that this tool would perform well with rare variants, as their genotypes will be clustered with higher frequency variants according to measured *X* and *Y* allele intensities (as opposed to *between-sample* methods, where rare variants are sparsely located in the heterozygous cluster).

To speed up the genotyping process, samples were called using *GenoSNP* as soon as they were released from the genotyping center. A posterior probability cutoff of 0.8 was used to achieve higher quality calls. To ascertain the quality of the results once all samples were genotyped, the mean and the median intensities of all calls for each sample were plotted.

#### optiCall

The *optiCall* tool uses a mixture of *between-* and *within-sample* models. It uses a subset of (*X*,*Y*) intensities from random samples at a random marker to find a prior distribution to the statistical model used to call genotypes across markers. This distribution is inferred by using an EM algorithm to fit a four-class mixture of Student’s *t*-distributions. The initial values used by the EM algorithm are obtained by using the *kmeans++* algorithm [17] and the individual genotype’s *a priori* probabilities are assumed to be uniform (0.25 for every cluster, including the outlier’s cluster). Then, a second mixture of *t*-distributions is used for the *between-sample* clustering, where the previously estimated *priors* are used in a Maximum-A-Posteriori (MAP) estimate of its parameters.

To measure the quality of genotype calls, *optiCall* relies on deviation from the Hardy-Weinberg Equilibrium (HWE). The tool will try to improve the genotype calls when the HWE test fails (*p*<5×10^-15^) by fitting the previously described model without a prior.

#### zCall

This tool functions as a post-processing tool (after a default one has been used) [14]. The *zCall* tool separates the clusters for rare variants by partitioning the (*X*,*Y*) intensity space using horizontal and vertical thresholds. Their positions are derived from the mean and variance of the homozygote clusters for common variants that were previously called and are scaled according to a *z-score* factor to optimize concordance with the default tool. Genotypes are then assigned with respect to their position relative to the *z-score* scaled coordinates. Accordingly, rare variants are called by defining a distance threshold. The homozygote threshold for the major allele is estimated from the first calling tool’s genotypes, and the rare allele’s threshold is estimated by linear regression from the means and standard deviations of *X* and *Y* intensities of common markers.

As recommended by the authors, *zCall* was used as a post-processing step after *GenCall* (*GenomeStudio*). Version 3.2 was used, where all *z* thresholds were derived from *GenomeStudio*’s final report, from which samples were filtered out based on call rate and global heterozygosity. A *z* threshold of 8 was used after comparing the concordance with the original calls (maximum of 99.27%). Only missing genotypes from the original *GenomeStudio* report were recalled by *zCall*.

### Dataset

The four tools were applied to a dataset consisting of 10,517 unique samples from the Montreal Heart Institute (MHI) Cohort. We also included 95 experimental replicates of *NA17281*, 15 replicates of *NA17251* and 3 replicates of *NA12763* from the Coriell Institute (the latter being sequenced by the *1000 Genomes Project*[18]). Finally, 93 and 40 MHI cohort samples were replicated 3 and 2 times, respectively. All 10,520 samples (10,856 including replicated ones) were genotyped using the Illumina HumanExome BeadChip, assessing 247,870 markers including 140 insertions/deletions (which were discarded from the present analysis). Some of these markers (214,599) are present in NHLBI GO Exome Sequencing Project (ESP) database [19], and 93% of these are rare variants with a minor allele frequency (MAF) below 5% in the European American population according to the ESP database [19]. The research protocol was approved by the Montreal Heart Institute research ethics review board and all participants signed an informed consent.

### Agreement between tools

We used Cohen’s kappa (*κ*) and Fleiss’ pi (*π*), two widely used statistics, to compute the extent of agreement between raters [20], or in this case, genotype calling tools. Cohen’s *κ* computes the extent of agreement between two tools by first computing the overall agreement probability (Equation 1), using a two-way contingency table (Additional file 1: Table S1), for the distribution of *n* samples by tools (rater) and genotype category, where *n*_*kl*_ indicates the number of samples that tool 1 and 2 classified into genotypes *k* and *l*, respectively.

(1) $$p_{a}=\overset{q}{\underset{k=1}{\sum }}\frac{n_{\text{kk}}}{n}$$

In a comparable contingency table, Cohen’s *κ* is estimated using Equation 2.

(2) $$\gamma_{\kappa }=\frac{p_{a}-p_{e|\kappa }}{1-p_{e|\kappa }},\text{where}\;\;p_{e|\kappa }=\overset{q}{\underset{k=1}{\sum }}\frac{n_{\text{Ak}}n_{\text{Bk}}}{n^{2}}$$

where *p*_*a*_ is the observed proportion of agreement (Equation 1) and *p*_*e*|*κ*_ is the proportion of agreement expected by chance.

The data can be summarized in a frequency table (Additional file 1: Table S2), where, for a given sample *i* and genotype *k*, *r*_*ik*_ represents the total number of tools that called genotype *k* for sample *i*. Fleiss’ *π* is then defined by Equation 3 [21].

(3) $${\hat{\gamma }}_{\pi }=\frac{p_{a}-p_{e|\pi }}{1-p_{e|\pi }}\;\left\{\begin{array}{ccc} p_{a} & = & \frac{1}{n}\overset{n}{\underset{i=1}{\sum }}\overset{q}{\underset{k=1}{\sum }}\frac{r_{\text{ik}}(r_{\text{ik}}-1)}{r(r-1)}\\ p_{e|\pi } & = & \overset{q}{\underset{k=1}{\sum }}{\hat{\pi }}_{k}^{2},\;\;\text{and}\;\;{\hat{\pi }}_{k}=\frac{1}{n}\overset{n}{\underset{i=1}{\sum }}\frac{r_{\text{ik}}}{r} \end{array}\right)$$

The possible set of genotypes included the *no call* genotype, as all tools might agree that a marker is impossible to be categorized in either of the three genotype clusters (homozygous or heterozygous) due to quality issues (*e.g.* low intensity).

### Error rates

Several methods for error rate estimation (*ϵ*) with pedigree data have been proposed and reviewed by Liu *et al.* for their use with unrelated samples [22]. The genotypic model (as defined in Equation 4) provides a proper estimation of the error rate and proposes different constraints on the parameter space in order to make the model parameters identifiable.

(4) $$\begin{array}{l} \epsilon =\frac{2(3C_{1}+3C_{3}-1)\pm \sqrt{{\left[\;6(C_{1}+C_{3})-4\right]}^{2}+12{\left(C_{1}-C_{3}\right)}^{2}}}{6}\\ \;\text{where}\;\left\{\begin{array}{ccc} C_{1} & ≃ & \frac{n_{\text{AA}}}{n}\\ C_{2} & ≃ & \frac{n_{\text{AB}}}{n}\\ C_{3} & ≃ & \frac{n_{\text{BB}}}{n} \end{array}\right) \end{array}$$

The genotypic model was tested by Liu *et al.* for common variants. However, we found that the possible values of *ϵ* were out of bounds (*i.e.* negative or above one) with the HumanExome BeadChip data where a majority of markers are rare. This can be explained by the proportion of the minor allele in the population, *p*_1_, which is almost null. For these cases, *ϵ* was approximated using *ϵ*≃(*C*_1_-*C*_3_+1)/3 (Additional file 1: Equation S1).

## Results and discussion

### Clustering quality

#### GenoSNP

**Figure 1:**
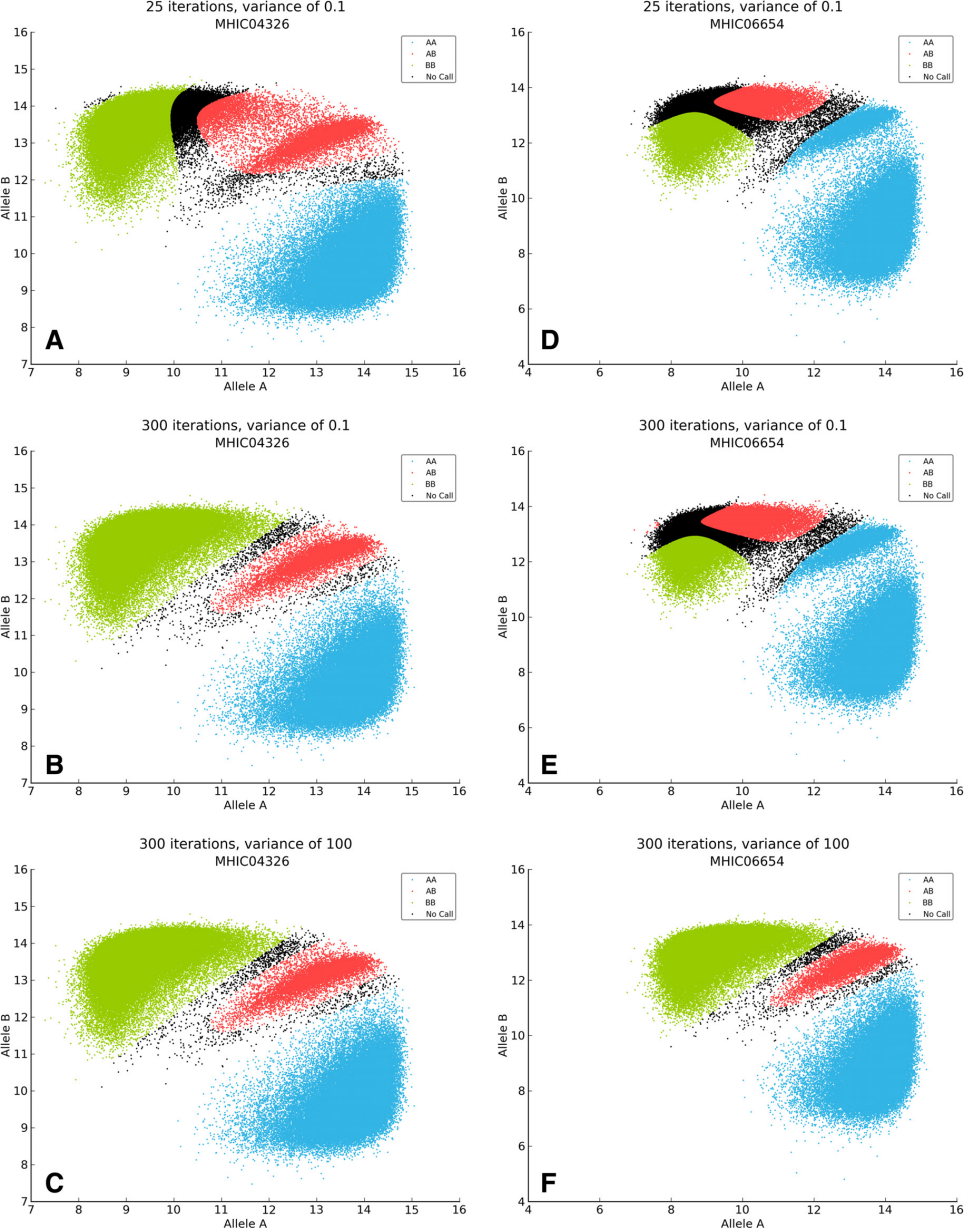
**Impact of parameters on GenoSNP’s clustering.** Impact of the choice of initial parameters on *GenoSNP*’s clustering of two samples (*MHIC04326* shown in **(A)**, **(B)** and **(C)**, and *MHIC06654* shown in **(D)**, **(E)** and **(F)**). The first row shows the clustering results of the two samples using the default parameters (25 EM iterations and an initial variance of 0.1). The second row shows the results when keeping an initial variance of 0.1, but increasing the number of EM iterations to 300. The last row shows the results when increasing both the initial variance and the number of EM iterations to 100 and 300, respectively. In all cases, only markers in the BeadPool 1 are kept. Each point represents the raw B allele intensities against the raw A allele intensities (base two logarithm in both cases) of each marker. The *AA*, *AB* and *BB* genotypes are shown in blue, red and green, respectively. Markers that were below the quality threshold of 0.8 are shown in black.

**Figure 2:**
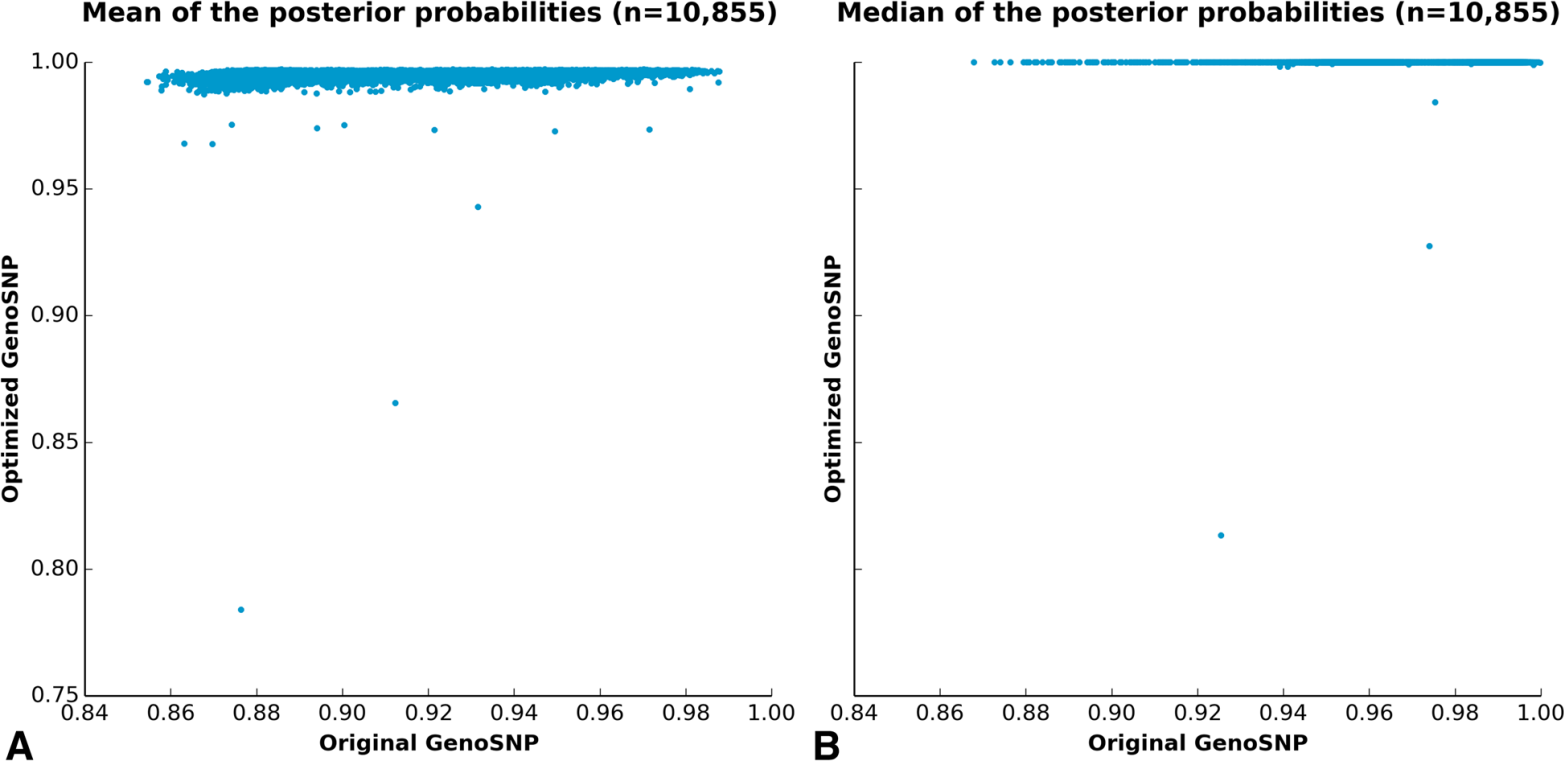
**GenoSNP clustering quality.** Quality assessment of *GenoSNP*’s clustering for original (*x* axis) against optimized (*y* axis) parameters. For each sample, both the **(A)** mean and the **(B)** median of the posterior probability of all markers are shown. Each point represents a sample. A good clustering has a mean and a median calling probability close to one.

The *GenoSNP* tool returns the probability of belonging to one of the three genotype clusters (homozygous *A*, *B* or heterozygous *AB*) for each evaluated genotype. The maximal probability is used to define the genotype to be called. We observed a majority of samples with a high proportion of low quality calls (close to the 0.8 quality threshold used). Cluster plots were created for some of the poorly performing samples which raised concern about the calling quality (Figure 1A and 1D). Suspecting a lack of convergence, modifications to the tool were made to increase the number of iterations of the VB-EM procedure. This improved the quality of the clustering for some of the samples (Figure 1B and 1E). Further modifications to the initial *X* and *Y* intensities’ variance parameter from 0.1 to 100 (in addition to the increased number of iterations) greatly improved the quality of the clustering tool (Figure 1C and 1F). To efficiently ascertain the effect of the optimized parameters on the calling quality, plots of the mean and the median of the maximal probability of each sample have been created (Figure 2A and 2B respectively), showing a net increase of the probabilities. To further improve the comparison between the original *GenoSNP* and the modified version (300 iterations and an initial *X* and *Y* intensities’ variance of 100), the two tools (original and optimized) were used for comparison with the other tools in this study.

#### zCall

According to Goldstein *et al.*[14], the optimal value of the *z* threshold should be determined by trying different values of *z* to find the one with the most concordance to the original *GenCall* calls. Here, the optimal value of *z* was determined to be 8, having a concordance of 99.27% with the original data.

### Missing rates

**Figure 3:**
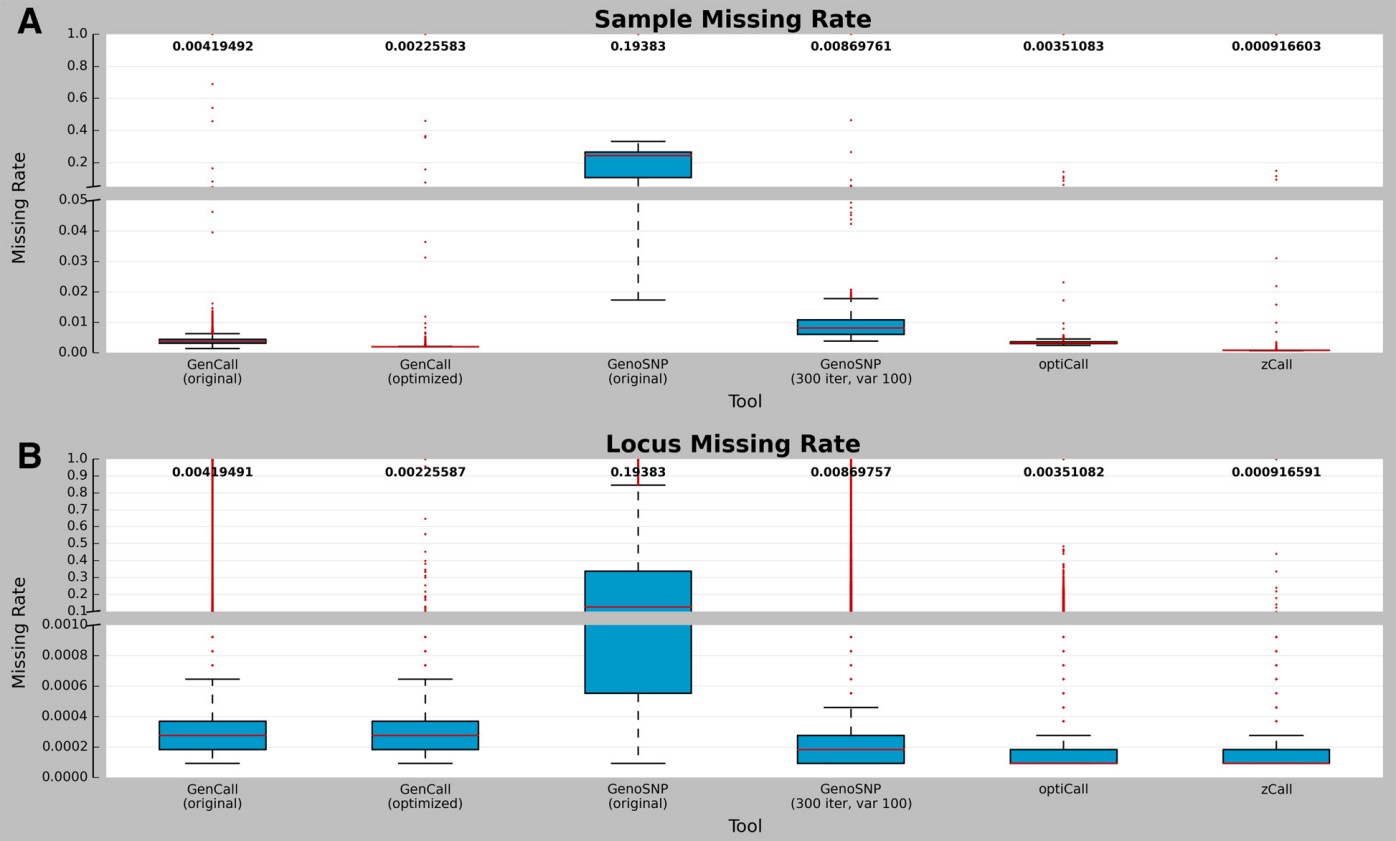
**Locus and sample missing rate.** Boxplots showing the distribution of **(A)** locus and **(B)** sample missing rates. Values closer to 0 (100% completeness) are preferable. Rates are shown for each of the six tools: *GenCall* (original and optimized cluster files), *GenoSNP* (original and optimized), *optiCall* (without excluding markers failing Hardy-Weinberg) and *zCall*. The median is shown with a red line and the mean is indicated above each plot. The red dots represent outliers according to the interquartile range. The rates are shown for 10,856 samples and 247,590 markers (markers located on chromosome Y were excluded).

One important property of a calling tool is its capacity to assign a genotype to the majority of samples and markers (*i.e.* the calling rate, or inversely, the missing rate). The sample and marker missing rates of the six tools were compared (Figure 3A and 3B respectively). The original version of *GenoSNP* had the highest missing rate (19% for both sample and marker). The optimized version of *GenoSNP* increased both the mean sample and marker calling rates by 18.5% (from 80.6% to 99.1%). These rates were inferior to those of the other tools: 99.6%, 99.8%, 99.6% and 99.9% for *GenCall* (original and optimized cluster file), *optiCall* and *zCall*, respectively. It is important to note that the missing rate of *zCall* is bound to be less than or equal to that of *GenCall*, as *zCall* will only call missing genotypes from the results produced by *GenCall*. Also, the missing rates of *GenCall* were slightly better when using the optimized cluster file when compared to the original one. A non-parametric *Friedman Rank Sum* test comparing tools showed a statistically significant difference in both sample and marker missing rates (p-value <2.2×10^-16^ in both cases), even though the rates seemed similar (Figure 3). Pairwise dependent-samples non-parametric sign tests comparing pairwise tools were also significant (p-value <2.2×10^-16^ in all cases). More specifically, 0.55- and 0.83-fold decreases, on average, were observed for sample and marker missing rates, respectively, when comparing the original and the optimized cluster file for *GenCall*.

### Precision estimates

**Figure 4:**
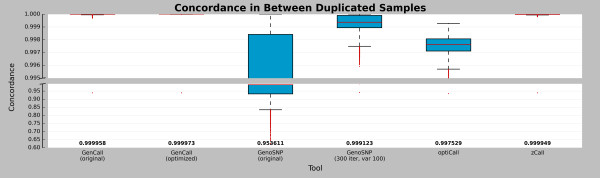
**Duplicated sample concordance.** Boxplots showing the distribution of pairwise concordance of duplicated samples. Red lines are the median and the numbers at the bottom of the plot represent the concordance means. The red dots represent the outliers according to the interquartile range. The six tools are shown (from left to right): *GenCall* (original and optimized cluster files), *GenoSNP* (original and optimized), *optiCall* (without excluding markers failing Hardy-Weinberg) and *zCall*. A total of 4,892 sample pairs is shown.

The dataset contained a high number of technical replicates. The concordance of genotype calls between replicates was computed for the five tools (Figure 4). The optimized version of *GenoSNP* increased concordance between sample duplicates from a mean of 95.361% to 99.912%, but did not exceed the performance of *GenCall* (means of 99.996% for both the original and the optimized cluster files). Even though *zCall* reduced the missing rate from a mean of 0.4% to 0.09%, it added variability in the newly called genotypes, slightly decreasing the concordance between replicated samples when compared with the original results (*GenCall*, original cluster file). Overall, the call concordance between replicates was similar (> 99% concordance). *GenCall* had the highest concordance rate with a mean of 99.997% between replicated sample pairs. Optimizing *GenCall*’s cluster file had only a minor effect on the mean concordance, with a difference of 1.5×10^-3^%.

### Accuracy estimates

One sample sequenced by the *1000 Genomes Project* was included three times to the original dataset to assess the concordance with next-generation sequencing. The genotypes called from the different tools were compared to the third release of the *1000 Genomes Project*, comprised of low coverage whole genome and high coverage exome sequencing data. The comparison with this gold standard was performed using the *pyGenClean* tool [23]. Since the majority of markers in the HumanExome BeadChip are located in exons, the results were mostly compared to the high coverage sequencing data (81.6% of the overlapping markers). Table 1 shows the concordance for the three replicates of *NA12763*. Apart from the original *GenoSNP*, all the tools had concordance rates greater than 99%. *GenCall* (optimized cluster file) had the highest concordance rate (mean of 99.897%), closely followed by *zCall* (mean of 99.879%). *GenCall* (original cluster file) also performed well, with a mean concordance of 99.855%. Then, calls were categorized according to their allele content: either (1) homozygous calls for the common allele or (2) involving the rare allele (heterozygous or homozygous calls for the rare allele). The frequencies were computed using the corresponding dataset (*i.e.* tool). Table 2 shows the concordance for the three replicates of *NA12763* for genotypes called as homozygous for the common allele. Apart from the original *GenoSNP* on one replicate, all had concordance rates greater than 99%. *GenCall* (original cluster file) had the highest concordance rate (mean of 99.948%), closely followed by *GenCall* (optimized cluster file) and *zCall* (mean of 99.939% and 99.928% respectively). Table 3 summarizes the concordance rates with the gold standard for genotypes involving the rare allele. *GenCall* (optimized cluster file) had the highest concordance rate (mean of 99.493%), closely followed by *zCall* (mean of 99.405%). The other tool had a lower concordance rate (*i.e.* between 95% and 97%).

**Table 1:** Call concordance with the 1000 Genomes Project (all calls)

**Tool**	**NA12763 _R**		**NA12763 _R1**		**NA12763 _R2**	
	**Rate**	**Number**	**Rate**	**Number**	**Rate**	**Number**
GenCall (original)	0.998591	127,062	0.998623	127,093	0.998425	126,980
GenCall (optimized)	0.998963	127,323	0.998963	127,323	0.998979	127,312
GenoSNP (original)	0.966771	113,365	0.907042	97,345	0.762193	93,555
GenoSNP (optimized)	0.996161	126,848	0.996446	126,893	0.995795	126,748
optiCall	0.995421	127,334	0.995413	127,323	0.994995	127,277
zCall	0.998785	127,545	0.998793	127,544	0.998785	127,545

Call concordance and number of compared markers for the three control replicates when compared to the *1000 Genomes Project*. The following six tools were compared: *GenCall* (original and optimized cluster files), *GenoSNP* (original and optimized), *optiCall* (without excluding markers failing Hardy-Weinberg) and *zCall*.

**Table 2:** Call concordance with the 1000 Genomes Project (homozygous common allele)

**Tool**	**NA12763 _R**		**NA12763 _R1**		**NA12763 _R2**	
	**Rate**	**Number**	**Rate**	**Number**	**Rate**	**Number**
GenCall (original)	0.999478	114,975	0.999461	115,008	0.999486	114,876
GenCall (optimized)	0.999393	115,288	0.999384	115,288	0.999393	115,279
GenoSNP (original)	0.999630	97,236	0.998973	78,868	0.925482	74,210
GenoSNP (optimized)	0.999190	114,814	0.999138	114,910	0.999207	114,687
optiCall	0.998990	114,815	0.998972	114,805	0.999006	114,701
zCall	0.999281	115,438	0.999281	115,437	0.999290	115,438

Call concordance and number of compared markers for the three control replicates when compared to the *1000 Genomes Project*. For each dataset (*i.e.* tool), only genotypes called as homozygous of the common allele (according to the allele frequency computed using the corresponding dataset) were kept for analysis. The following six tools were compared: *GenCall* (original and optimized cluster files), *GenoSNP* (original and optimized), *optiCall* (without excluding markers failing Hardy-Weinberg) and *zCall*.

**Table 3:** Call concordance with the 1000 Genomes Project (heterozygous and homozygous rare allele)

**Tool**	**NA12763 _R**		**NA12763 _R1**		**NA12763 _R2**	
	**Rate**	**Number**	**Rate**	**Number**	**Rate**	**Number**
GenCall (original)	0.990155	12,087	0.990650	12,085	0.988351	12,104
GenCall (optimized)	0.994848	12,035	0.994931	12,035	0.995014	12,033
GenoSNP (original)	0.767847	16,179	0.514147	18,590	0.137233	19,507
GenoSNP (optimized)	0.967032	12,042	0.970397	11,992	0.963052	12,071
optiCall	0.962401	12,527	0.962478	12,526	0.958045	12,585
zCall	0.994053	12,107	0.994136	12,107	0.993970	12,107

Call concordance and number of compared markers for the three control replicates when compared to the *1000 Genomes Project*. For each dataset (*i.e.* tool), only genotypes called as heterozygous or homozygous of the rare allele (according to the allele frequency computed using the corresponding dataset) were kept for analysis. The following six tools were compared: *GenCall* (original and optimized cluster files), *GenoSNP* (original and optimized), *optiCall* (without excluding markers failing Hardy-Weinberg) and *zCall*.

### Inter-tool agreement

**Figure 5:**
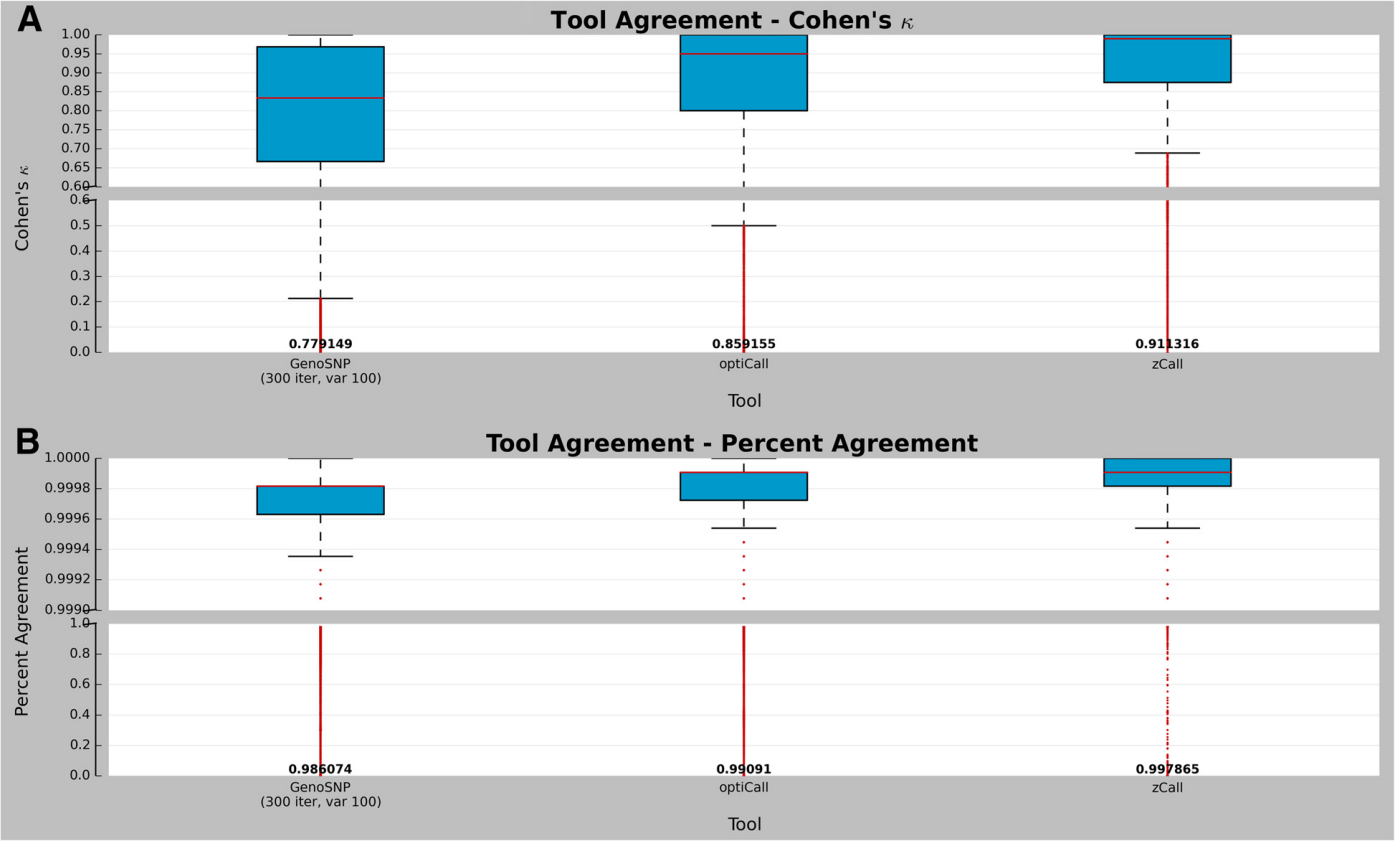
**Pairwise tool agreement.** Boxplots showing the Cohen’s *κ* coefficient **(A)** and the percent agreement **(B)** for all calls when *GenCall* is compared with the following three tools: *GenoSNP* (optimized), *optiCall* (without excluding markers failing Hardy-Weinberg) and *zCall*. Red lines are median and the numbers at the bottom of the plot represent the concordance means. The red dots represent the outliers according to the interquartile range.

**Figure 6:**
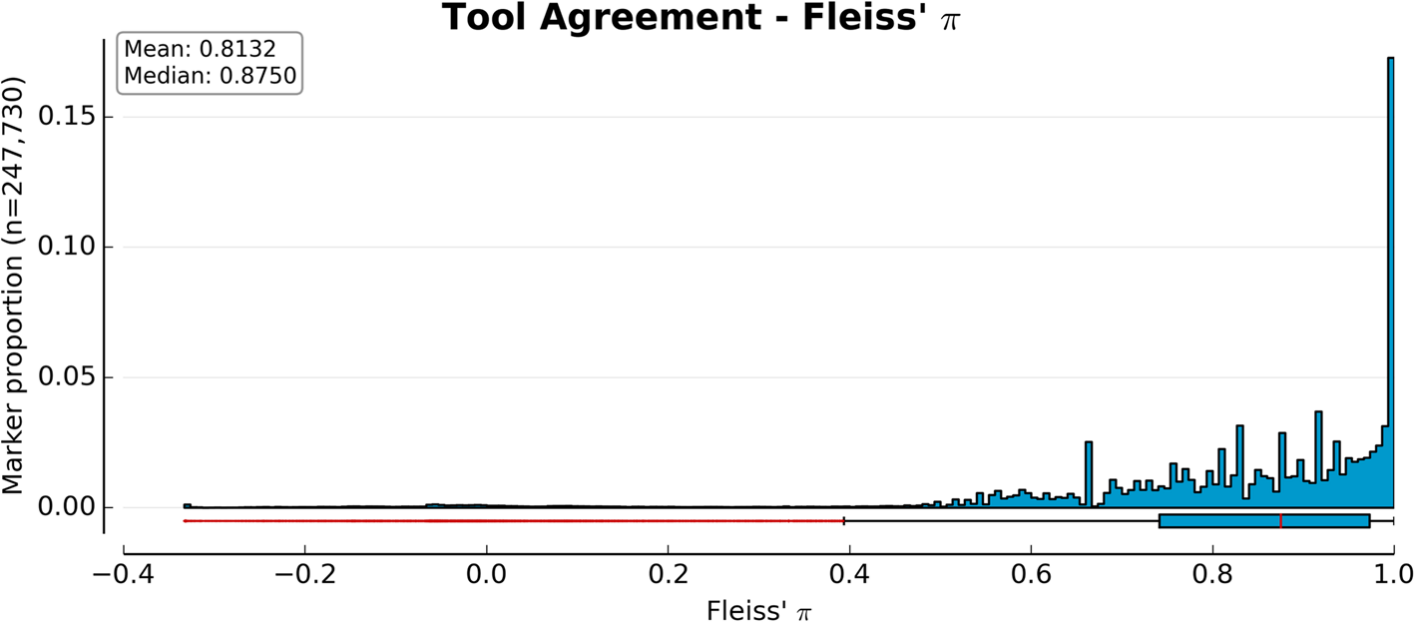
**Tool agreement.** Distribution of Fleiss’ *π* coefficient for all 247,730 markers when the following four tools are compared: *GenCall* (optimized cluster file), *GenoSNP* (optimized), *optiCall* (without excluding markers failing Hardy-Weinberg) and *zCall*. Values closer to 1 indicate good agreement. Using the interquartile range, there were a total of 12,122 outliers (represented as red dots in the boxplot).

To estimate tool agreement, three coefficients were computed: Cohen’s *κ* and percent agreement (both shown in Figure 5), and Fleiss’ *π* (Figure 6). Since *GenCall* (using the optimized version of the cluster file) provided the best result set, its agreement with the other three tools (excluding the original version of *GenoSNP*) was considered (Figure 5A and B). The best agreement was between *GenCall* and *zCall*, as expected due to the dependence of *zCall* on the results of *GenCall*. Both metrics showed a high number of outliers according to the interquartile range dispersion^b^. To assess the overall agreement between the four tools (excluding *GenCall* using the original cluster file and the original version of *GenoSNP*), Fleiss’ *κ* was computed (Figure 6). The agreement was fairly good between the tools when comparing all markers (common and rare). There was a total of 12,122 outliers according to the interquartile range dispersion, a majority of which (91.3%) were rare variants (MAF <1%, according to frequencies computed using *GenCall* results) and 5% were markers that were zeroed out while optimizing *GenCall*’s cluster file. The proportion of rare variants (when compared to common or zeroed out ones) was significantly higher in the outlier group (Wald statistic, *p*<0.001; logistic regression). Out of the approximately three thousands outlier markers that were comparable with the *1000 Genomes Project* data, *GenCall* had the highest concordance for the three replicates of *NA12763* (average of 98.925%), followed by *zCall* (average of 98.448%) (Additional file 1: Table S3).

### Error rate estimates

**Figure 7:**
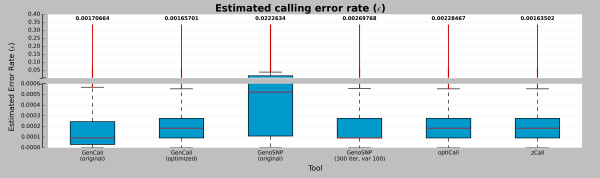
**Marker error rate distribution.** Boxplots showing the marker error rate estimation for each the six tools: *GenCall* (original and optimized cluster files), *GenoSNP* (original and optimized), *optiCall* (without excluding markers failing Hardy-Weinberg) and *zCall*. The numbers at the top of the plot represent the mean of the error rate distribution and the red lines are the median of each distribution. Red dots represent the outliers according to the interquartile range. Values above 1/3 were discarded since it’s not plausible to have such a large error rate in practice [22].

Error rates were estimated by using the genotypic model (Liu *et al.*[22]), where the six tools were evaluated (Figure 7). Except for the original version of *GenoSNP*, all tools showed a comparable estimated error rate mean (approximately 0.002). The *zCall* tool provided the lowest estimated error rate with a mean of 0.164%, followed by *GenCall* (optimized cluster file) with a mean of 0.166%. A non-parametric *Friedman Rank Sum* test comparing tools showed a significant difference in the error rate distributions (p-value <2.2×10^-16^ in both cases). Pairwise dependent-samples non-parametric sign tests comparing pairwise tools were also significant (p-value <2.2×10^-16^ in all cases).

## Conclusions

This study compares the performance of widely used clustering tools when applied to genotyping data from Illumina’s HumanExome BeadChip. This genotyping array includes a large proportion of rare variants that were previously identified by sequencing technologies. The dataset used here included 10,520 unique samples along with a high number of technical replicates for quality assessment.

Contrary to our original expectations, *GenoSNP*, which relies on a *within-sample* model, did not perform well when used straight “out of the box”. This might be explained by the high density of markers in each BeadPool, which is higher than for Illumina’s Human 1M BeadChip (comprising an overall higher number of markers). The latter was successfully tested by *GenoSNP*’s authors. This problem was mostly obvious with cluster plots (Figure 1) and with graphs showing the summarized quality of the calls per sample (Figure 2). The concordance of results from the original *GenoSNP* tool with the *1000 Genomes Project*, however, remained high (mean of 87.87%). The optimized tool (modified initial variance and number of iterations) increased the concordance to a mean of 99.61%. The quality threshold of 0.8 provided a better separation of the three clusters, but increased the missing rate of both sample and marker (mean of 0.87%).

*GenCall* relies on a *between-sample* model that requires reference parameters to perform its clustering. As such, it is common practice to manually modify the reference cluster parameters to ensure the best quality of results when the population analyzed is different from the one used to generate the reference parameters. This task requires a significant amount of manual labor which increases with the number of samples and markers. Loading the raw data, normalizing and modifying the original cluster file took one person 5 work days, compared to only a few days to generate the intensity files for the other tools. When we compared the genotype results from *GenCall* using the original cluster file with the ones generated with the optimized cluster file, we saw only limited improvement in the overall concordance of genotype calls with those of the gold standard. When partitioning calls according to their allele content, we saw a limited decrease in the concordance of homozygous calls involving the common allele when compared with the gold standard, while the concordance of calls involving the rare allele improved by approximately 0.5%. A limited improvement in the concordance between technical replicates was observed. However, the optimization of the cluster file had a greater impact on the missing rate per sample and per loci (0.19% improvement in both cases). According to normal quality control procedures, a filter to remove samples and markers with a missing rate greater than 2% is typically imposed prior to genetic analysis [23]. By optimizing the cluster file, an average of 2,963 markers (1.2%) per sample could be rescued by lowering the marker missing rate below the 2% quality threshold.

It should be noted that this study was limited by the lack of an independent gold standard for concordance analysis. *GenCall*’s (and *zCall*’s) concordance with the *1000 Genomes Project* might be overestimated due to the nature of its reference parameters. Indeed, the reference cluster file was created by using estimated cluster position for each of the markers in the HapMap population. Hence, the sample *NA12763* is expected to have a higher concordance value than samples from the Montreal Heart Institute Cohort owing to the CEU HapMap population. One possible way to overcome this limitation would be to sequence a few of our own cohort samples, however, it could be argued that the sequencing technology itself would not provide an adequate gold standard in this situation.

Many study designs will plan to include experimental replicates chosen from the genotyped cohort to assess the reproducibility of the genotyping pipeline and the precision of the results. Including a reference sample to the study design offers the additional possibility of assessing the accuracy of the results. High precision is particularly important as it provides optimal power for statistical analysis [24] and can prevent type I errors due to plate biases or subgroup effects [25]. *GenCall* (using the original or the optimized cluster file) provided the highest concordance rate between experimental replicates (99.997%) when compared to other tools. The other tools (except for the original *GenoSNP*) performed relatively well, all providing a mean concordance greater than 99%.

All clustering tools had a high accuracy (>99.8%) when calling common markers (except for *GenoSNP* at only one of the three replicates of *NA12763*). Other metrics have shown that all the tools (once optimized) performed almost at the same efficiency level on the HumanExome BeadChip. The major difference arose when in the presence of rare markers, where the accuracy of all the tools decreased below 99.5% (as low as 96% for some) for genotypes involving the rare allele. *Within-sample* tools like *GenoSNP* can process samples in an efficient manner by running single samples and smaller batches of samples in parallel instead of having to wait for a large amount of samples to be genotyped and normalized. Unfortunately, even with the proper optimization of initial parameters, *GenoSNP* could not outperform the other tools. However, *GenCall*, Illumina’s proprietary tool, performed better than the other tested tools with respect to concordance with the gold standard for genotypes involving the rare allele (accuracy) and slightly better for the concordance in between technical replicates (precision). The fact that *zCall* has been run as a post-process of *GenCall* without the use of the reference cluster file optimization (since it is not mandatory) might explain why it’s accuracy was not as high as the optimized *GenCall* for calls involving the rare allele. Since the third version of *zCall* derives its thresholds from *GenomeStudio*’s report, the call rate will increase and better accuracy might be possible if the original cluster file is optimized beforehand.

Recommending a single clustering tool according to the metrics shown in this report is not straightforward. In general, *GenCall* (optimized cluster file) outperformed the other tools in terms of precision and accuracy (overall, and for the calls involving the rare allele). Its accuracy was also higher for the markers with low inter-tool agreement. However, when using the optimized cluster file, *GenCall*’s accuracy for the homozygous calls (common allele) was lower than when using the default cluster file. When considering missing and estimated error rates, *zCall* outperformed the other tools, closely followed by *GenCall* (optimized cluster file). It is important to mention that the task of optimizing the cluster file is time consuming. Furthermore, all the other tools presented here require intensity data provided by the *GenomeStudio* software and possible file conversion, which increase the total execution time.

The parallel use of multiple clustering tools offers the possibility of identifying discordant markers which can be further investigated. But notably, the manual optimization of *GenCall*’s cluster file at those loci and the visual inspection of the cluster plots should provide high quality datasets for downstream analysis.

## Endnotes

^a^*θ* values of the center of the AA and BB clusters in normalized polar coordinates, respectively.

^b^ Outliers are observations that fall below *Q*_1_-1.5(*IQR*) or above *Q*_3_+1.5(*IQR*), where *Q*_1_ and *Q*_3_ are respectively the first and third quartiles and *IQR*=*Q*_3_-*Q*_1_ is the interquartile range.

## Competing interests

The authors declare that they have no competing interests.

## Authors’ contributions

LPLP worked on the experimental design, optimized *GenoSNP*’s initial parameters, performed analyses, compared the tools, implemented the statistical tests and drafted the manuscript. MAL contributed to the analysis and to the manuscript. AB contributed to the statistical analyses and the manuscript. SP contributed to the experimental design, analysis and the manuscript. VN contributed to the manual optimization of *GenCall* and the manuscript. JCT leads the Montreal Heart Institute Cohort and directs the genomic project with the HumanExome BeadChip. MPD supervised the project, participated in the design and coordination of the study, supervised the laboratory and revised the manuscript. All authors read and approved the final manuscript.

## Supplementary Material

Additional file 1 **Additional materials. Supplemental Equation S1:** The genotypic model for error rate estimation was tested by Liu *et al.* for common variants only. However, we found that the possible values of *ϵ* were out of bound (*i.e.* negative or above one) for a majority of rare markers. For those cases, *ϵ* was approximated using *ϵ*≃(*C*_1_-*C*_3_+1)/3. **Supplemental Table S1:** Distribution of *n* samples by calling tool in *q* categories. The set of possible categories are all possible genotypes (*i.e.q*∈{AA,AB,BB,00}, where 00 represents the *no call* category). This table is computed for each marker and for each pair of calling tools. The overall agreement probability and Cohen’s *κ* are shown in Equation 1 and 2 of the main text, respectively. **Supplemental Table S2:** Distribution of *r* calling tools by *n* samples and *q* response categories. The set of possible categories are all possible genotypes (*i.e.q*∈{AA,AB,BB,00}, where 00 represents the *no call* category). This table is computed for each marker and for each calling tool. Fleiss’ *π* is explained in Equation 3 of the main text. **Supplemental Table S3:** Call concordance and number of compared markers for the three control replicates when compared to the *1000 Genomes Project* for the markers that were outliers for their Fleiss’ *π* values. The following four tools were compared: *GenCall* (optimized cluster file), *GenoSNP* (optimized), *optiCall* (without excluding markers failing Hardy-Weinberg) and *zCall*. Click here for file

## References

[B1] Hindorff LA MacArthur J Morales J Junkins HA Hall PN Klemm AK Manolio TA **A Catalog of Published Genome-Wide Association Studies** 2013 [http://www.genome.gov/gwastudies]

[B2] The International HapMap Consortium **A haplotype map of the human genome** Nature 2005 437 7063 1299 1320 10.1038/nature04226 16255080 PMC1880871

[B3] The International HapMap Consortium **A second generation human haplotype map of over 3.1 million SNPs** Nature 2007 449 7164 851 861 10.1038/nature06258 17943122 PMC2689609

[B4] The International HapMap 3 Consortium **Integrating common and rare genetic variation in diverse human populations** Nature 2010 467 7311 52 58 10.1038/nature09298 20811451 PMC3173859

[B5] Manolio TA Collins FS Cox NJ Goldstein DB Hindorff LA Hunter DJ McCarthy MI Ramos EM Cardon LR Chakravarti A Cho JH Guttmacher AE Kong A Kruglyak L Mardis E Rotimi CN Slatkin M Valle D Whittemore AS Boehnke M Clark AG Eichler EE Gibson G Haines JL Mackay TF McCarroll SA Visscher PM **Finding the missing heritability of complex diseases** Nature 2009 461 7265 747 753 10.1038/nature08494 19812666 PMC2831613

[B6] Zuk O Hechter E Sunyaev SR Lander ES **The mystery of missing heritability: genetic interactions create phantom heritability** Proc Natl Acad Sci 2012 109 4 1193 1198 10.1073/pnas.1119675109 22223662 PMC3268279

[B7] Kaiser J **Genetic influences on disease remain hidden** Science 2012 338 6110 1016 1017 10.1126/science.338.6110.1016 23180835

[B8] llumina Inc **Data Sheet: Human Exome BeadChips** 2013 [http://res.illumina.com/documents/products/datasheets/datasheet_humanexome_beadchips.pdf]

[B9] Ritchie ME Liu R Carvalho BS Irizarry RA Australia and New Zealand Multiple Sclerosis Genetics Consortium (ANZgene) **Comparing genotyping algorithms for Illumina’s Infinium whole-genome SNP BeadChips** BMC Bioinformatics 2011 12 68 10.1186/1471-2105-12-68 21385424 PMC3063825

[B10] Giannoulatou E Yau C Colella S Ragoussis J Holmes CC **GenoSNP: a variational Bayes within-sample SNP genotyping algorithm that does not require a reference population** Bioinformatics 2008 24 19 2209 2214 10.1093/bioinformatics/btn386 18653518

[B11] Li G Gelernter J Kranzler HR Zhao H **M3: an improved SNP calling algorithm for Illumina BeadArray data** Bioinformatics 2012 28 3 358 365 10.1093/bioinformatics/btr673 22155947 PMC3268244

[B12] Shah T Liu J Floyd J Morris J Wirth N Barrett J Anderson C **optiCall: a robust genotype-calling algorithm for rare, low-frequency and common variants** Bioinformatics 2012 28 12 1598 1603 10.1093/bioinformatics/bts180 22500001 PMC3371828

[B13] llumina Inc **GenomeStudio Software v2011.1 Release Notes** 2011 [http://support.illumina.com/documents/MyIllumina/1f2d00ae-b759-489b-82f9-0163ce085ae7/GenomeStudio_2011.1_ Release_Notes.pdf]

[B14] Goldstein JI Crenshaw A Carey J Grant GB Maguire J Fromer M O’Dushlaine C Moran JL Chambert K Stevens C Sklar P Hultman CM Purcell S McCarroll SA Sullivan PF Daly MJ Neale BM Swedish Schizophrenia Consortium, ARRA Autism Sequencing Consortium **zCall: a rare variant caller for array-based genotyping Genetics and population analysis** Bioinformatics 2012 28 19 2543 2545 10.1093/bioinformatics/bts479 22843986 PMC3463112

[B15] Maher B **The case of the missing heritability** Nature 2008 456 7218 18 21 10.1038/456018a 18987709

[B16] Eichler EE Flint J Gibson G Kong A Leal SM Moore JH Nadeau JH **Missing heritability and strategies for finding the underlying causes of complex disease** Nat Rev Genet 2010 11 6 446 450 10.1038/nrg2809 20479774 PMC2942068

[B17] Arthur D Vassilvitskii S **k-means++: the advantages of careful seeding** Proceedings of the Eighteenth Annual ACM-SIAM Symposium on Discrete Algorithms, SODA ’07 2007 Philadelphia: Society for Industrial and Applied Mathematics 1027 1035 [http://dl.acm.org/citation.cfm?id=1283383.1283494]

[B18] Abecasis GR Auton A Brooks LD DePristo MA Durbin RM Handsaker RE Kang HM Marth GT McVean GA 1000 Genomes Project Consortium **An integrated map of genetic variation from 1,092 human genomes** Nature 2012 491 7422 56 65 10.1038/nature11632 23128226 PMC3498066

[B19] NHLBI GO Exome Sequencing Project (ESP) **Exome Variant Server** 2013 [http://evs.gs.washington.edu/EVS/]

[B20] Gwet KL **Computing inter-rater reliability and its variance in the presence of high agreement** Br J Math Stat Psychol 2008 61 29 48 10.1348/000711006X126600 18482474

[B21] Fleiss JL **Measuring nominal scale agreement among many raters** Psychol Bull 1971 76 5 378 382

[B22] Liu N Zhang D Zhao H **Genotyping error detection in samples of unrelated individuals without replicate genotyping** Hum Hered 2009 67 3 154 162 10.1159/000181153 19077433 PMC2782542

[B23] Lemieux Perreault LP Provost S Legault MA Barhdadi A Dubé MP **pyGenClean: efficient tool for genetic data clean up before association testing** Bioinformatics 2013 29 13 1704 1705 10.1093/bioinformatics/btt261 23652425 PMC3694635

[B24] Powers S Gopalakrishnan S Tintle N **Assessing the impact of non-differential genotyping errors on rare variant tests of association** Hum Hered 2011 72 3 153 160 10.1159/000332222 22004945 PMC3214826

[B25] Mayer-Jochimsen M Fast S Tintle NL **Assessing the impact of differential genotyping errors on rare variant tests of association** PloS One 2013 8 3 e56626 10.1371/journal.pone.0056626 23472072 PMC3589406

