# Supplementary material for: Comparison of genotype clustering tools with rare variants
Source: BMC Bioinformatics. 2014 Feb 21;15:52. doi: 10.1186/1471-2105-15-52 (PMC3941951; doi:10.1186/1471-2105-15-52)
Supplement: Additional file 1 — Additional materials. Supplemental Equation S1: The genotypic model for error rate estimation was tested by Liu et al. for common variants only. However, we found that the possible values of ϵ were out of bound (i.e. negative or above one) for a majority of rare markers. For those cases, ϵ was approximated using ϵ≃(C1-C3+1)/3. Supplemental Table S1: Distribution of n samples by calling tool in q categories. The set of possible categories are all possible genotypes (i.e.q∈{AA,AB,BB,00}, where 00 represents the no call category). This table is computed for each marker and for each pair of calling tools. The overall agreement probability and Cohen’s κ are shown in Equation 1 and 2 of the main text, respectively. Supplemental Table S2: Distribution of r calling tools by n samples and q response categories. The set of possible categories are all possible genotypes (i.e.q∈{AA,AB,BB,00}, where 00 represents the no call category). This table is computed for each marker and for each calling tool. Fleiss’ π is explained in Equation 3 of the main text. Supplemental Table S3: Call concordance and number of compared markers for the three control replicates when compared to the 1000 Genomes Project for the markers that were outliers for their Fleiss’ π values. The following four tools were compared: GenCall (optimized cluster file), GenoSNP (optimized), optiCall (without excluding markers failing Hardy-Weinberg) and zCall. [file 1471-2105-15-52-S1.pdf]

Comparison of genotype clustering tools with rare variants

Additional Materials

Lemieux Perreault L.-P., Legault M.-A., Barhdadi A., Provost S., Normand V., Tardif J.-C. and Dubé M.-P.

Supplemental Equation 1 - Error rate for rare markers

The genotypic model for error rate estimation was tested by Liu *et al.* for common variants only. However, we found that the possible values of ϵ were out of bound (*i.e.* negative or above one) for a majority of rare markers. For those cases, ϵ was approximated using $\epsilon \simeq (C_1 - C_3 + 1)/3$, as described below.

$$
\begin{aligned}
C_1 &= p_1^2(1-2\epsilon) + 2p_1p_2\epsilon + p_2^2\epsilon && \text{(S1)}\\
C_3 &= p_1^2\epsilon + 2p_1p_2\epsilon + p_2^2(1-2\epsilon) && \text{(S2)}\\
C_1 - C_3 &= p_1^2(1-2\epsilon) + 2p_1p_2\epsilon + p_2^2\epsilon - p_1^2\epsilon - 2p_1p_2\epsilon - p_2^2(1-2\epsilon)\\
&= p_1^2(1-2\epsilon) + p_2^2\epsilon - p_1^2\epsilon - p_2^2(1-2\epsilon)\\
&= p_1^2 - 2p_1^2\epsilon + p_2^2\epsilon - p_1^2\epsilon - p_2^2 + 2p_2^2\epsilon\\
&= p_1^2 - 3p_1^2\epsilon + 3p_2^2\epsilon - p_2^2\\
&= (p_1^2 - p_2^2) - 3(p_1^2 - p_2^2)\epsilon\\
&= (1-3\epsilon)(p_1^2 - p_2^2)\\
&= (1-3\epsilon)(p_1 - p_2)(p_1 + p_2)\\
&= (1-3\epsilon)(p_1 - (1-p_1))\\
C_1 - C_3 &= (1-3\epsilon)(2p_1 - 1) && \text{(S3)}\\
2p_1 - 1 &= \frac{C_1 - C_3}{1-3\epsilon}\\
2p_1 &= \frac{C_1 - C_3}{1-3\epsilon} + 1\\
p_1 &= \frac{1}{2}\left(\frac{C_1 - C_3}{1-3\epsilon}\right) + \frac{1}{2} && \text{(S4)}\\
\text{if } p_1 \approx 0 &\Rightarrow \frac{1}{2}\left(\frac{C_1 - C_3}{1-3\epsilon}\right) + \frac{1}{2} \approx 0\\
&\Rightarrow \frac{C_1 - C_3}{1-3\epsilon} + 1 \approx 0\\
&\Rightarrow C_1 - C_3 + 1 - 3\epsilon \approx 0\\
&\Rightarrow C_1 - C_3 + 1 \approx 3\epsilon\\
&\Rightarrow \epsilon \approx \frac{C_1 - C_3 + 1}{3} && \text{(S5)}
\end{aligned}
$$

Supplemental Table 1 - Overall agreement probability and Cohen's κ calculation

Table S1: Overall agreement probability and Cohen's κ calculation. Distribution of n samples by calling tool in q categories. The set of possible categories are all possible genotypes (*i.e.* $q \in \{\mathrm{AA}, \mathrm{AB}, \mathrm{BB}, 00\}$, where 00 represents the *no call* category). This table is computed for each marker and for each pair of calling tools. The overall agreement probability and Cohen's κ are shown in Equation 1 and 2 of the main text, respectively.

	**Tool B**				
**Tool A**	1	2	$\cdots$	q	*Total*
1	n_{11}	n_{12}	$\cdots$	n_{1q}	n_{A1}
2	n_{21}	n_{22}	$\cdots$	n_{2q}	n_{A2}
$\vdots$			$\cdots$		$\vdots$
q	n_{q1}	n_{q2}	$\cdots$	n_{qq}	n_{Aq}
Total	n_{B1}	n_{B2}	$\cdots$	n_{Bq}	n

Supplemental Table 2 - Fleiss' π calculation

Table S2: Fleiss' π calculation. Distribution of r calling tools by n samples and q response categories. The set of possible categories are all possible genotypes (*i.e.* $q \in \{\mathrm{AA}, \mathrm{AB}, \mathrm{BB}, 00\}$, where 00 represents the *no call* category). This table is computed for each marker and for each calling tool. Fleiss' π is explained in Equation 3 of the main text.

	**Category**				
**Sample**	1	2	$\cdots$	q	*Total*
1	r_{11}	r_{12}	$\cdots$	r_{1q}	r
2	r_{21}	r_{22}	$\cdots$	r_{2q}	r
$\vdots$			$\cdots$		$\vdots$
n	r_{n1}	r_{n2}	$\cdots$	r_{nq}	r
Total	r_{+1}	r_{+2}	$\cdots$	r_{+q}	nr

Supplemental Table 3 - Call concordance with the 1000 Genomes Project (Fleiss' π outliers)

Table S3: Call concordance with the *1000 Genomes Project* (Fleiss's π outliers). Call concordance and number of compared markers for the three control replicates when compared to the *1000 Genomes Project* for the markers that were outliers for their Fleiss' π values. The following four tools were compared: *GenCall* (optimized cluster file), *GenoSNP* (optimized), *optiCall* (without excluding markers failing Hardy-Weinberg) and *zCall*.

Tool	NA12763_R		NA12763_R1		NA12763_R2	
	Rate	Number	Rate	Number	Rate	Number
GenCall (optimized)	0.989157	3,228	0.989151	3,226	0.989434	3,218
GenoSNP (optimized)	0.895096	3,079	0.908626	3,130	0.878186	3,021
optiCall	0.851575	3,207	0.849688	3,200	0.830272	3,158
zCall	0.984485	3,416	0.984485	3,416	0.984485	3,416
